# Comparative evaluation of isavuconazonium sulfate, voriconazole, and posaconazole for the management of invasive fungal infections in an academic medical center

**DOI:** 10.1186/s12941-019-0311-3

**Published:** 2019-03-20

**Authors:** Edward T. Van Matre, Shelby L. Evans, Scott W. Mueller, Robert MacLaren, Douglas N. Fish, Tyree H. Kiser

**Affiliations:** 1 Department of Clinical Pharmacy and Translational Science, University of Tennessee Health Science Center College of Pharmacy, Memphis, TN USA; 2 Department of Pharmacy, University of Colorado Health Memorial Hospital, Colorado Springs, CO USA; 3 Department of Clinical Pharmacy, University of Colorado Skaggs School of Pharmacy and Pharmaceutical Sciences, Aurora, CO USA; 4 Department of Clinical Pharmacy, University of Colorado Anschutz Medical Campus, 12850 East Montview Blvd, mailstop C238, Aurora, CO 80045 USA

**Keywords:** Isavuconazole, Voriconazole, Posaconazole, Mycoses, Aspergillus

## Abstract

**Background:**

Invasive fungal infections are a major cause of morbidity and mortality. Newer antifungals may provide similar efficacy with improved safety compared to older more established treatments. This study aimed to compare clinically relevant safety and efficacy outcomes in real world patients treated with isavuconazole, voriconazole, or posaconazole.

**Methods:**

This single center retrospective matched cohort study evaluated adults between January 2015 and December 2017. The primary outcome was a composite safety analysis of antifungal related QTc prolongation, elevated liver function tests (> 5 times ULN), or any documented adverse drug event. Key secondary outcomes included: individual safety events, 30-day readmissions, magnitude of drug interactions with immunosuppressive therapy, and overall cost.

**Results:**

A total of 100 patients were included: 34 patients in the voriconazole group and 33 patients within each of the isavuconazole and posaconazole groups. The composite safety outcome occurred in 40% of the total cohort and was different between isavuconazole (24.2%), voriconazole (55.9%), and posaconazole (39.4%; p = 0.028). Change in QTc (p < 0.01) and magnitude of immunosuppression dose reduction (p = 0.029) were different between the three groups. No differences in mortality, length of stay, readmission, or infection recurrence were observed between groups (p > 0.05 for all). The overall medication cost, when including therapeutic drug monitoring, was not different between treatments (p = 0.36).

**Conclusions:**

Patients treated with isavuconazole resulted in fewer composite safety outcomes, driven by decreased incidence of QTc prolongation, compared to patients treated with voriconazole or posaconazole. Overall drug cost was not significantly different between the treatment therapy options.

## Background

Invasive fungal infections are a major cause of morbidity and mortality among patients, particularly those with a compromised immune system. According to published data, invasive aspergillosis is the most common type of fungal infection in stem cell transplant patients and the second most common fungal infection in solid organ transplant patients, with an incidence of 19% during a surveillance period from 2001 to 2006 [1, 2]. The 1-year survival rate associated with invasive aspergillosis infections is roughly 59% in solid organ transplant patients and 25% among stem cell transplant patients [1]. Mucormycosis was identified as the third most common cause of fungal infections in stem cell transplant patients and while rarer than invasive aspergillosis infections, the all-cause mortality rate in these patients is estimated at 54% [3].

Invasive mucormycosis and aspergillosis are difficult infections to treat and the cost of therapy adds additional burden to the United States healthcare system. In 2009, it was estimated that each case of mucormycosis results in an average cost of $97,743, totaling over $50 million per year [4]. Monitoring of therapeutic drug levels for certain medications further adds to this already high treatment cost. Current first-line treatment for invasive aspergillosis, based on the Infectious Disease Society of America guidelines, is voriconazole. The primary alternative therapy options are isavuconazole and liposomal or lipid formulations of amphotericin B. Posaconazole is a third-line agent for patients with clinical failure or adverse events. Echinocandin therapy may be considered as combination therapy with a triazole or as salvage therapy [5]. Current first-line treatment for invasive mucormycosis is amphotericin B, in patients who can tolerate the nephrotoxic side effects. Other options include posaconazole and isavuconazonium sulfate (isavuconazole) for alternate or step-down therapy [6].

In recent clinical trials isavuconazole showed similar efficacy and improved safety compared to voriconazole for the treatment of invasive molds and mucormycosis [7, 8]. The use of isavuconazole in a variety of clinical settings is expanding. This study aimed to evaluate the utilization of isavuconazole at a single center academic medical center, and to comparatively evaluate effectiveness, safety, and cost between isavuconazole, voriconazole, and posaconazole.

## Methods

### Study design

This was a retrospective cohort study conducted at the University of Colorado Hospital between January 2015 and December 2017. The study was reviewed and approved by the Colorado Multiple Institutional Review Board. Patients were identified through the University of Colorado Hospital’s electronic medical records using medication usage reports. Study data were collected and managed using REDCap electronic data capture tools hosted at the University of Colorado Anschutz Medical Campus [9]. Patients were included within the study if they were ≥ 18 years of age and received isavuconazonium sulfate (isavuconazole), voriconazole, or posaconazole for active treatment of confirmed or suspected fungal infection as part of routine clinical care. Patients were excluded if they were vulnerable subjects (e.g. prisoners, pregnancy) as defined by institutional review board.

### Primary end point

The primary outcome evaluated was a composite safety outcome (occurrence of any of the following safety events after the initiation of an antifungal): QTc prolongation (> 470 ms for females and > 450 ms for males), liver function tests five times the upper limit of normal (ALT > 260 units/L and AST > 195 units/L), or any documented antifungal treatment related adverse event based on primary team documentation within the electronic medical record.

### Secondary end points

The secondary outcomes evaluated were the individual components of the composite outcome, percent change in QTc length from baseline; percent change in calcineurin inhibitor serum concentration from baseline using the following equation:

$$\frac{{\left( {\frac{Baseline\,total\,daily\,dose}{Baseline\,steady\,state\,serum\,concentration}} \right) - \left( {\frac{Adjusted\,total\,daily\,dose}{Adjusted\,steady\,state\,serum\,concentration}} \right)}}{{\left( {\frac{Baseline\,total\,daily\,dose}{Baseline\,steady\,state\,serum\,concentration}} \right)}}\, \times \,100$$

total cost of inpatient antifungal therapy per day, utilizing average wholesale price (AWP) and hospital cost for therapeutic drug monitoring; all-cause in-hospital mortality; hospital length of stay; and intensive care unit length of stay.

### Statistical analysis

In order to minimize selection bias and ensure similar comparator groups, patients were grouped based on indication for antifungal use, concomitant disease processes, and baseline demographics (e.g. age, neutropenia, renal dysfunction, and hepatic dysfunction). Similar frequency of each group were included. Based on previously reported adverse event rates of approximately 60% [7], 30 patients were needed in each group to detect a 30% difference in the primary outcome between groups with 80% power and an alpha of 5%.

Over the entire study period 35 patients received isavuconazole, 53 patients received posaconazole, and 115 patients received voriconazole. Given the disparity of use between the groups over the study period patients were matched to provide a balanced comparative evaluation. Patients were primarily matched on indication of use (treatment vs prophylaxis), followed by admission diagnosis and treatment month. The isavuconazole treatment group was used as the reference for patient matching.

Categorical data were compared utilizing Chi squared test, if statistical significance was found pairwise comparisons were performed between the groups. Continuous normally distributed data were compared utilizing analysis of variance test followed by pairwise t-test, and non-normally distributed data were compared utilizing Kruskal–Wallis test followed by pairwise Mann–Whitney U tests. All tests were two sided with an α level of 5% and were performed utilizing JMP^®^, Version 14 (SAS Institute Inc., Cary, NC).

Given the changing prescribing patterns at the University of Colorado Hospital and transition of use away from posaconazole a post hoc analysis was performed comparing a combined group of patients who received isavuconazole or voriconazole to patients receiving posaconazole for the previously stated primary and secondary outcomes. In this analysis categorical variable were compared utilizing Chi squared test. Continuous normally distributed data was compared using t-test and non-normally distributed data was compared utilizing Mann–Whitney U tests.

## Results

A total of 100 patients were included within the study, 34 patients were included within the voriconazole group and 33 patients were included within the isavuconazole and posaconazole groups respectively. Of the 100 patients included within the study a majority were male (54%) with a mean age of 55.9 years of age and a majority of patients (62%) were admitted to UCH with a primary oncologic diagnosis. Voriconazole was utilized more frequently as the primary treatment option (82.4%, p ≤ 0.001) and isavuconazole was initiated more frequently for refractory treatment (39.4%, p = 0.027) or intolerance, as defined by the prescribing clinician, to other medications (27.3%, p = 0.011). The presumed clinical diagnosis based on clinical laboratory values (galactomannan assays and β-d-glucan assays), radiologic imaging, and culture data was similar between the three treatment groups (p = 0.101) and is outlined in Table 1. Pretreatment acute kidney injury or renal dysfunction was the documented reason for isavuconazole initiation for 32.4% of patients. Patients included within the isavuconazole treatment group had a higher mean baseline serum creatinine (1.57 ± 1.09, p = 0.002) and mean baseline QTc (478 ± 46, p = <0.001) when compared to the voriconazole and posaconazole groups. Complete baseline characteristics and indications for use are reported in Table 1.

**Table 1:** Baseline characteristics

Characteristic	Total (n = 100)	Isavuconazole (n = 33)	Voriconazole (n = 34)	Posaconazole (n = 33)	P-value
Age, years	55.9 ± 13.7	58.8 ± 14.0	56.9 ± 11.4	52.1 ± 15.1	0.124
Female, n (%)	46 (46)	16 (48.5)	14 (41.2)	16 (48.5)	0.785
Baseline Dialysis, n (%)	7 (7)	3 (9.1)	2 (5.9)	2 (6.1)	0.852
Baseline Serum Creatinine, mg/dL	1.23 ± 0.82	1.57 ± 1.09	0.88 ± 0.42	1.24 ± 0.66	0.002
Primary diagnosis of Oncology, n (%)	62 (62)	17 (51.5)	23 (67.6)	22 (66.7)	0.097
Hematologic Malignancy, n (%)	58 (93.6)	16 (94.1)	22 (95.6)	20 (90.9)	0.806
Primary diagnosis of Solid Organ Transplant, n (%)	26 (26)	14 (42.4)	5 (14.7)	7 (21.2)	0.097
Baseline AST, units/L	27.3 ± 36.0	22.0 ± 17.7	21.9 ± 15.5	36.4 ± 41.8	0.053
Baseline ALT, units/L	26.8 ± 28.2	22.2 ± 22.0	22.5 ± 22.1	37.1 ± 53.3	0.160
Baseline QTc, milliseconds	457 ± 40	478 ± 46	445 ± 29	450 ± 35	0.001
Concurrent echinocandin therapy, n (%)	23 (23)	8 (24.2)	5 (14.7)	10 (30.3)	0.298
Concurrent QTc prolonging medications, n (%)	83 (83)	24 (72.7)	28 (82.35)	31 (93.9)	0.057
Concurrent Immunosuppression, n (%)	52 (52)	20 (60.6)	17 (50.0)	15 (45.4)	0.447
Formal infectious disease team consultation, n (%)	71 (71)	28 (84.8)	20 (58.8)	23 (69.7)	0.055
Indication for use					
Primary, n (%)	58 (58)	10 (30.3)	28 (82.4)	20 (60.6)	< 0.001
Refractory, n (%)	24 (24)	13 (39.4)	4 (11.8)	7 (21.2)	0.027
Intolerance, n (%)	16 (16)	9 (27.3)	1 (2.9)	6 (18.2)	0.011
Other, n (%)	2 (2)	1 (3.0)	1 (2.9)	0 (0)	0.444
Treatment diagnosis					
Zygomycosis, n (%)	8 (8.33)	3 (9.1)	0 (0.0)	5 (15.2)	0.101
Aspergillosis, n (%)	30 (31.2)	12 (36.4)	10 (33.3)	8 (24.2)	
Empiric treatment, n (%)	58 (60.4)	18 (54.6)	20 (66.7)	20 (60.6)	

The primary composite safety outcome occurred in 40% of the total cohort and was different between isavuconazole (24.2%), voriconazole (55.9%), and posaconazole (39.4%; p = 0.028). The secondary analysis of the individual components of the primary composite outcome demonstrated there was not a significant difference in incidence of LFT elevation (p = 0.876), or incidence of adverse reactions (p = 0.356). There was a reduced incidence of QTc prolongation in the isavuconazole group compared to the other two groups (p = 0.037). There was not a significant difference in hospital mortality (p = 0.878) or hospital length of stay (p = 0.515) between the groups. Complete clinical outcomes data is presented within Table 2. The overall cost of the drug and utilized therapeutic drug monitoring was not different between the three groups (p = 0.360). Complete cost outcomes are represented within Table 3.

**Table 2:** Clinical outcomes

Characteristic	Total (n = 100)	Isavuconazole (n = 33)	Voriconazole (n = 34)	Posaconazole (n = 33)	P-value
Composite safety, n (%)	40 (40)	8 (24.2)	19 (55.9)	13 (39.4)	0.028
QTc prolongation following drug initiation, n (%)	28 (28)	4 (12.1)	13 (38.2)	11 (33.3)	0.037
LFT elevation, n (%)	8 (8.0)	2 (6.1)	3 (8.8)	3 (9.1)	0.876
Adverse reaction, n (%)	9 (9)	2 (6.1)	5 (15.2)	2 (6.1)	0.356
Change in QTc, milliseconds	7.5 ± 42.0	− 18.0 ± 37.6	20.5 ± 37.8	22.6 ± 38.6	0.001
Max QTc, milliseconds	464.2 ± 35.1	460.0 ± 29.5	465.4 ± 33.8	467.4 42.2	0.739
Change in ALT, units/L	93.2 ± 393.6	95.1 ± 440.3	105.6 ± 448.5	78.5 ± 281.2	0.964
Change in AST, units/L	192.4 ± 894.7	159.5 ± 717.3	259.2 ± 1226.2	155.4 ± 629.8	0.875
Duration of inpatient therapy, days	12.9 ± 15.3	11.9 ± 11.9	12.6 ± 10.8	14.3 ± 21.5	0.809
Mortality, n (%)	48 (48)	15 (45.5)	16 (47.1)	17 (51.5)	0.878
ICU length of stay, days	12.7 ± 37.1	10.0 ± 15.0	11.1 ± 23.2	17 ± 58.7	0.718
Hospital length of stay, days	32.9 ± 37.4	31.8 ± 25.9	28.2 ± 19.5	38.7 ± 56.7	0.515
30 day readmission, n (%)	29 (42)	8 (34.78)	7 (30.4)	14 (60.9)	0.077
Recurrent infection, n (%)	9 (14.1%)	1 (4.5%)	4 (19.1%)	4 (19.1%)	0.230
Percent change in immunosuppression dose	− 46.1 [− 27.8, − 57.8]	− 34.3 [− 4.04, − 46.5]	− 48.4 [− 35.1, − 65.9]	− 46.4 [− 35.1, − 65.9]	0.029

**Table 3:** Cost outcomes

Characteristic	Total (n = 100)	Isavuconazole (n = 33)	Voriconazole (n = 34)	Posaconazole (n = 33)	P-value
Total drug cost, $	3912 [2499; 6635]	3570 [2476; 6458]	3891 [3013; 4904]	4798 [2546; 8011]	0.360
Total drug cost per day, $	478 [369; 625]	500 [396; 625]	369 [302; 474]	624 [485; 787]	< 0.001
Therapeutic drug monitoring, n (%)	22 (22)	0 (0)	14 (41.2)	8 (24.2)	< 0.001
Total cost, $	4032 [2499; 6703]	3571 [2476; 6458]	4011 [3013; 4904]	4798 [2546; 8131]	0.360

Medians with IQR

Pairwise comparisons for the primary composite safety outcome demonstrated fewer safety events in the isavuconazole group compared to the voriconazole group (p = 0.008). No differences for the primary composite safety outcome were observed when comparing vorizonazole and posaconazole groups (p = 0.224). The isavuconazole group was found to have decreased incidence of QTc prolongation when compared to the voriconazole group (p = 0.015) and the posaconazole group (p = 0.043), and a significant difference was not seen between the voriconazole and posaconazole groups (p = 0.676). Similarly, the QTc from baseline was decreased in the isavuconazole group when compared to the voriconazole group (p = <0.001) and the posaconazole group (p = <0.001). Voriconazole cost per day in drug cost was reduced compared to the isavuconazole group (p = <0.001) and the posaconazole group (p = <0.001), and the isavuconazole group’s cost was less per day than the posaconazole group (p = 0.015). Overall, when considering both total drug cost and therapeutic drug monitoring cost there was no difference between the three treatment groups (p = 0.360).

A post hoc analysis comparing a combined group of patients who received isavuconazole or voriconazole to patients receiving posaconazole was performed for the previously stated primary and secondary outcomes. The primary composite safety outcome was not different between the combined isavuconazole or voriconazole group when compared to the posaconazole group (40.3% vs 39.4%, p = 0.931). There was also no significant differences between these two groups regarding mortality (46.3% vs 51.5%, p = 0.622), duration of antifungal therapy (9 [5, 14] days vs 9 [3, 15.5] days, p = 0.774), ICU length of stay (2 [0, 10] days vs 3 [0, 11] days, p = 0.982), hospital length of stay (25 [15, 39] days vs 23 [14, 42.5] days, p = 0.608), total drug cost ($3787 [$2499; $5188] vs $7798 [$2546; $8011), p = 0.155), and total cost of therapy ($3787 [$2499; $5188] vs $4798 [$2546; $8131], p = 0.157). Differences were seen between the isavuconazole or voriconazole treatment when compared to the posaconazole treatment group in 30-day readmission rates (32.6% vs 60.9%, p = 0.038) and total drug cost per day of treatment ($409 [$340, $534] vs $624 ($624 [$485, $787] p = <0.001).

## Discussion

This study provides a real word evaluation of the extended triazole antifungal agents commonly utilized for the treatment and prophylaxis of invasive fungal infections. The primary finding of the study was a significant reduction of incidence of the composite safety outcome of QTc prolongation, liver function test five times the upper limit of normal and documented adverse drug reactions with the use of isavuconazole when compared to the use of voriconazole or posaconazole. When evaluating the pairwise comparisons the driving comparison was the reduction in the primary composite outcome between isavuconazole and voriconazole where there was a greater than 30% difference in occurrence rate. When evaluating each of the components of the primary outcome there were no differences found between the three antifungals for LFT elevation or documented adverse reactions. However, QTc prolongation occurred at a higher rate in the voriconazole group and the posaconazole group when compared to the isavuconazole treatment group. The significant differences in QTc prolongation was the primary driver in the overall composite outcome. This is particularly notable given the significantly longer QTc at baseline in the isavuconazole group compared to the voriconazole and posaconazole groups. Additionally, the overall change in QTc duration form baseline was significantly lower in the isavuconazole group with an 18.0 ± 37.6 ms decrease compared to a 20.5 ± 37.8 ms increase in the voriconazole group and a 22.6 ± 38.6 ms increase in the posaconazole group. Given the retrospective nature of this study QTc measurements were based on clinical orders for electrocardiograms and were not collected at prespecified time points. The reduction in QTc length has been demonstrated in previous studies [10, 11] likely due to inhibition of L-type calcium channels [12] not seen in other triazole antifungal agents, and is a clinical consideration of utilizing isavuconazole.

Statistically significant differences were not seen in the other reported clinical outcomes across the three groups particularly mortality, duration of therapy, and recurrent infection rates. This is similar to previously reported studies including prospective evaluations of aspergillosis and mucormycosis [7, 8]. In the post hoc analysis the combined group of voriconazole or isavuconazole had a lower 30-day readmission rate (32.6%) than the posaconazole treatment group (60.9%). Readmission rates of almost double for the posaconazole group may indicate the utilization of either isavuconazole or voriconazole provides a more sustained clinical treatment compared to posaconazole.

Within the study cohort baseline serum creatinine was significantly higher in the isavuconazole group (1.57 ± 1.09) and had a higher rate [n=3 (9.1%)] of patients on dialysis. This is likely due to concerns for sulfobutylether-beta-cyclodextrin sodium (SBECD) accumulation and toxicity in patients with a creatinine clearance < 50 ml/min. SBECD toxicity has not been reported in humans and recent data demonstrates SBECD from intravenous treatment doses of voriconazole and posaconazole is effectively removed via continuous veno-venous hemofiltration [13, 14]. The VITAL study, a single-arm open-label trial that assessed patients with invasive aspergillosis and renal dysfunction and patients with rare invasive fungal diseases, demonstrated similar results to a matched retrospective cohort treated with amphotericin B formulations and a favorable side effect profile [8]. Given this data, isavuconazole is frequently chosen as first line therapy for patients being treated for invasive mold infections with renal dysfunction. Additionally, isavuconazole is likely to be utilized in patients requiring broad intravenous antifungal therapy with poor renal function who do not require or are not candidates for renal replacement therapy.

Therapeutic drug monitoring was performed in 22% of the overall cohort. Therapeutic drug monitoring was utilized in 41.2% of the voriconazole group, 24.2% in the posaconazole group, and was not utilized within the isavuconazole treatment group. Recent data has demonstrated there is a high incidence of subtherapeutic plasma concentrations in patients receiving voriconazole and posaconazole treatment [15]. This is especially important for posaconazole suspension which has demonstrated high variability in target plasma concentration achievement [15]. Due to the variability plasma concentration therapeutic drug monitoring is necessary for voriconazole and posaconazole, particularly in patients with known invasive mold infections. Given the tolerability, efficacy [7], and lower patient plasma concentration variability [16] of isavuconazole the utility of therapeutic drug monitoring is currently unknown.

The percent change in immunosuppression dosage requirements indicates clinically significant effects on drug metabolism and elimination. There was a clinical and statistical difference in immunosuppressive drug dose alterations as isavuconazole group had a 34.3% [4.04, 46.5] decrease in immunosuppressive drug dosing compared to the voriconazole treatment group which required a 48.4% [35.1, 65.9] dose decrease and the posaconazole group which required a 46.4% [35.1, 65.9] dose decrease. This data shows true in-hospital clinical effects requiring clinical interventions due to known drug-drug interactions [17, 18].

There were no statistically significant differences in either drug cost or overall total cost of therapy between the three groups. However, the median total drug cost for the isavuconazole and voriconazole treatment groups were approximately $1000 less per patient than the posaconazole group. The drug cost when normalized per day of therapy was less in both the isavuconazole group and the voriconazole group compared to the posaconazole group.  The voriconazole group cost less per day than the isavuconazole therapy group. Isavuconazole does not require therapeutic drug monitoring and therefore had none reported during the study. The favorable pharmacokinetic drug profile is likely why there were more patients with baseline elevated serum creatinine compared to the other treatment groups [19]. The drug cost contributed approximately 97% of the total cost when evaluated across all groups and minimizes the overall cost impact of therapeutic drug monitoring. Our study did not account for clinical costs attributed to the interpretation and implementation of the results of the therapeutic drug monitoring in the voriconazole and posaconazole groups.

While this study does provide real world comparative data regarding the use of azole antifungals in invasive fungal infections, it does have several limitations. Given the retrospective nature of the study, there may be an inherent bias for the initial selection of azole antifungals for treatment, which was evident in baseline characteristic differences between the groups. The study was also not designed to evaluate for outpatient treatment or effects following discharge outside of 30-day readmission. Given the single center nature of the study, clinical practice and regional fungal biome differences may limit the external validity of the data. While this study helps to provide baseline comparisons of treatment options for invasive fungal infections, pragmatic prospective studies are needed to illuminate the roles each of the broad spectrum azole antifungals play in the treatment of invasive fungal diseases.

## Conclusions

Among patients treated for known or presumed invasive fungal infections at the University of Colorado Hospital, patients treated with isavuconazole resulted in fewer composite safety outcomes compared to patients treated with voriconazole or posaconazole driven by a lower incidence of QTc prolongation. There was no difference seen in treatment efficacy, patient clinical outcomes, or total treatment cost.

## Abbreviations

AWP: average wholesale price
SBECD: sulfobutylether-beta-cyclodextrin sodium
